# Polychlorinated biphenyls reduce the kinematics contractile properties of embryonic stem cells-derived cardiomyocytes by disrupting their intracellular Ca^2+^ dynamics

**DOI:** 10.1038/s41598-018-36333-z

**Published:** 2018-12-17

**Authors:** Paola Rebuzzini, Estella Zuccolo, Cinzia Civello, Lorenzo Fassina, Juan Arechaga, Amaia Izquierdo, Pawan Faris, Maurizio Zuccotti, Francesco Moccia, Silvia Garagna

**Affiliations:** 1 Laboratorio di Biologia dello Sviluppo, Dipartimento di Biologia e Biotecnologie, Università degli Studi di Pavia, Pavia, Italy; 2 Centre for Health Technologies (C.H.T.), Università degli Studi di Pavia, Pavia, Italy; 3 Laboratorio di Fisiologia Generale, Dipartimento di Biologia e Biotecnologie, Università degli Studi di Pavia, Pavia, Italy; 4 Dipartimento di Ingegneria Industriale e dell’Informazione, Università degli Studi di Pavia, Pavia, Italy; 5 Laboratory of Stem Cells, Development and Cancer, Department of Cell Biology and Histology, Faculty of Medicine and Nursing, Universidad del País Vasco, Vizcaya, Spain; 6 Department of Biology, College of Science, Salahaddin University, Erbil, Kurdistan-Region of Iraq Iraq

## Abstract

Persistent organic pollutants are a group of chemicals that include polychlorinated biphenyls (PCBs). PCBs exposure during adult life increases incidence and severity of cardiomyopathies, whereas *in utero* exposure determines congenital heart defects. Being fat-soluble, PCBs are passed to newborns through maternal milk, impairing heart functionality in the adult. It is still unknown how PCBs impair cardiac contraction at cellular/molecular levels. Here, we study the molecular mechanisms by which PCBs cause the observed heart contraction defects, analysing the alterations of Ca^2+^ toolkit components that regulate contraction. We investigated the effect that Aroclor 1254 (Aroclor), a mixture of PCBs, has on perinatal-like cardiomyocytes derived from mouse embryonic stem cells. Cardiomyocytes, exposed to 1 or 2 µg/ml Aroclor for 24 h, were analyzed for their kinematics contractile properties and intracellular Ca^2+^ dynamics. We observed that Aroclor impairs cardiomyocytes contractile properties by inhibiting spontaneous Ca^2+^ oscillations. It disrupts intracellular Ca^2+^ homeostasis by reducing the sarcoplasmic reticulum Ca^2+^ content and by inhibiting voltage-gated Ca^2+^ entry. These findings contribute to the understanding of the molecular underpinnings of PCBs-induced cardiovascular alterations, which are emerging as an additional life-threatening hurdle associated to PCBs pollution. Therefore, PCBs-dependent alteration of intracellular Ca^2+^ dynamics is the most likely trigger of developmental cardiac functional alteration.

## Introduction

Persistent organic pollutants are a variegated group of chemicals that include polychlorinated biphenyls (PCBs), the latter compounds containing a high number of chlorine atoms. PCBs have been widely used in electrical and electronic industries (production of plastics, adhesives, paints, carbonless copying paper, newsprint and caulking compounds^1,2^), since when, in 1979, their production and use were internationally banned. However, due to their high chemical stability, PCBs are still persisting and have wide diffusion into the environment.

Growing amounts of data link their bioaccumulation through the diet with their high toxicity; more specifically, several studies have evidenced that PCBs exposure during adult life leads to an increase in the incidence and severity of cardiomyopathies^3–7^. PCBs exposure alters the expression of GATA-4, Nkx-2.5, MEF-2c, OCT-1 cardiac nuclear transcription factors and other heart-specific genes (atrial and brain natriuretic peptide, alpha- and beta-myosin heavy chain, alpha-cardiac and alpha-skeletal actin) in adult primary rat cardiomyocytes^8^. In guinea pig ventricular myocytes, PCB 19 exposure decreases contractile force, action potential duration and amplitude, and intracellular Ca^2+^ transients through inhibition of L-type voltage-gated Ca^2+^ channels (VGCCs﻿)^9^. Also, in other adult cardiac cellular models, PCBs alter the expression and/or activity of several components of the intracellular Ca^2+^ signalling machinery^10^, such as type 2 ryanodine receptors (RyR2)^11^ and the Sarco-Endoplasmic-Reticulum Ca^2+^-ATPase (SERCA)^12^.

PCBs prenatal exposure through placenta determines congenital heart defects^13,14^ not attributable to inherited genetic mutations^5^. To this regard, exposed avian embryos display extensive cardiac dilation, thinner ventricle walls and reduced responsiveness to chronotropic stimuli^15^, whereas zebrafish embryos show heart defects, reduction of the heart beating rate and irregular and weak contractions^16^. Reduced heart size and functional deficits have been described in mouse foetuses, as well as cardiac hypertrophy in offsprings, the latter showing increased sensitivity to cardiovascular insults in adulthood^5^.

Importantly, being fat-soluble, PCBs accumulate in fat tissues and are passed to newborns through maternal milk^17^, increasing the myocardial wall thickness and inducing cardiac hypertrophy, thus impairing heart functionality in the adult^18^. Whilst the detrimental effects on the perinatal heart anatomy are evident, unknown are the PCBs effects at the cellular and molecular levels. Our study aims at dissecting the key physiological processes behind the observed heart contraction defects, by analyzing in perinatal cardiomyocytes the calcium-induced calcium-release mechanism (Ca^2+^ toolkit) that finely regulates contraction.

Perinatal-like cardiomyocytes (hereafter named cardiomyocytes) were obtained from the *in vitro* differentiation of mouse embryonic stem cells (mESCs). Functionally, these cardiomyocytes form compact beating syncytia and exhibit electrophysiological features of excitation-contraction coupling described for isolated perinatal cardiac cells^19–21^.

Following differentiation, cardiomyocytes were exposed for 24 h to Aroclor 1254 (Aroclor), a mixture of more than 80 PCBs isomers and congeners with high number of chlorine atoms (54%)^2^, at doses in the range of environmental contamination (1 and 2 µg/ml), then their kinematics contractile properties and the intracellular Ca^2+^ homeostasis were evaluated.

## Results

### Aroclor reduces the kinematics and dynamics properties (contractile properties) of beating syncytia

For the evaluation of the effects of Aroclor on the contractile properties of beating syncytia, we measured their kinematics and dynamics features on AVI videos, recorded from CTR samples and after 24 h exposure to 1 or 2 µg/ml Aroclor. The chronotropic (beat frequency [Hz]), inotropic (contraction force [pixel/s^2^] and contractility, i.e. the maximum contraction velocity, [pixel/s]) and ergotropic (consumption of ATP for kinetic energy [pixel^2^/s^2^]) features were mathematically calculated from the movement of the beating syncytia^22^.

**Figure 1:**
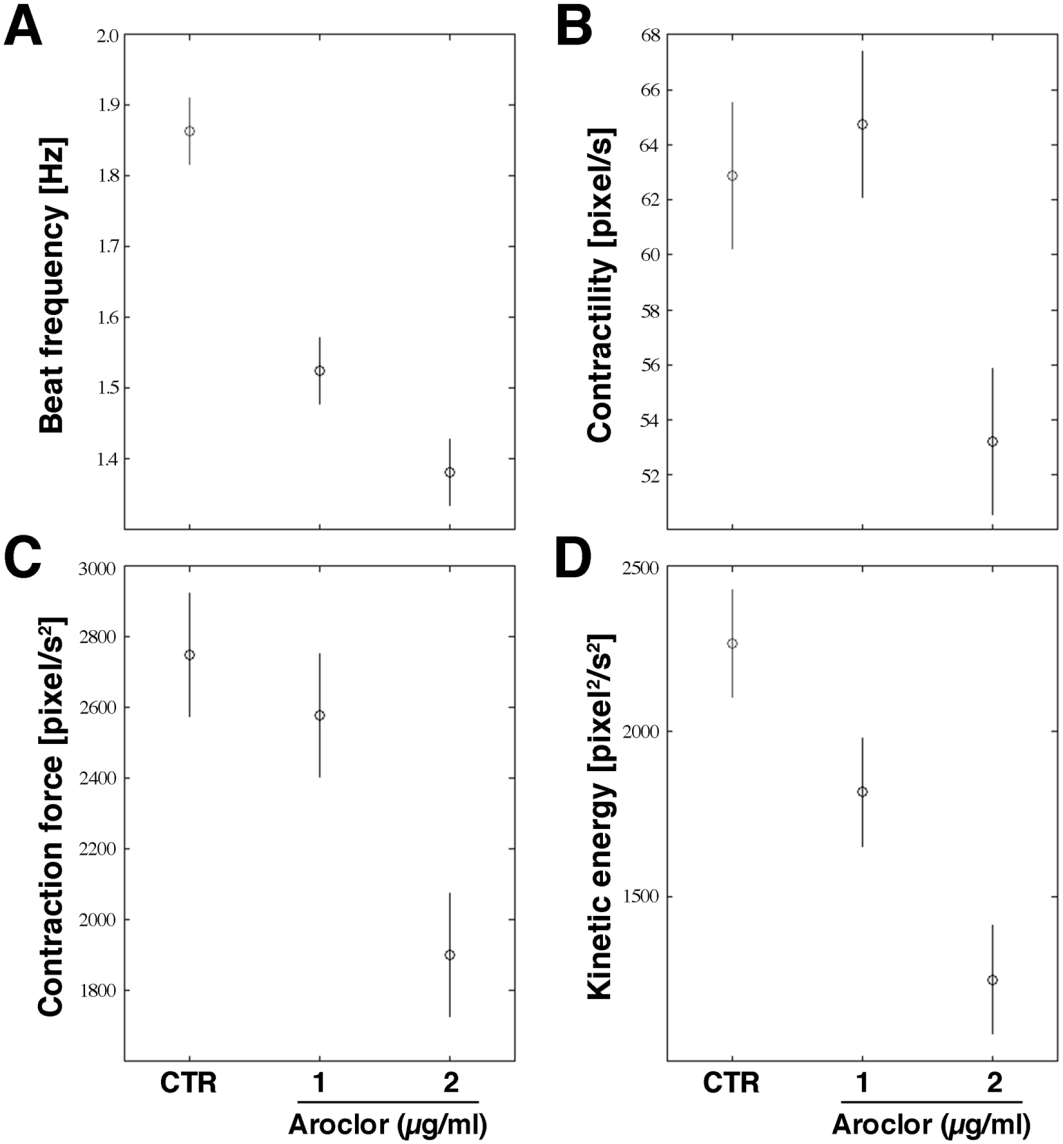
Kinematics contractile properties of cardiac beating syncytia. (**A**) Beat frequency [Hz]. (**B**) Contractility (maximum contraction velocity) [pixel/s]. (**C**) Contraction force [pixel/s^2^]. (**D**) Kinetic energy [pixel^2^/s^2^]. Horizontal bars represent the 95% confidence intervals for the differences between means according to the Least Significant Difference statistical test.

After exposure to 1 µg/ml Aroclor, both beat frequency and kinetic energy showed a 1.2-fold reduction (*p* < 0.05), whereas inotropic parameters remain unaltered (*p* > 0.05) when compared to CTR (Fig. 1A,D). A more adverse effect was observed when syncytia were exposed to 2 µg/ml, since all four parameters were significantly reduced (*p* < 0.05). Specifically, when compared to CTR, a 1.4-, 1.2-, 1.4-, 1.8-fold decrease of beat frequency, contractility, contraction force and kinetic energy, respectively, was observed (Fig. 1 and videos supplementary materials).

### Aroclor reduces spontaneous intracellular Ca^2+^ oscillations

Aroclor, as previously shown for other PCBs^23,24^, may induce alterations in the cardiomyocyte contraction properties through a derangement of the intracellular Ca^2+^ signalling machinery. Importantly, the contractile properties of beating syncytia depend on spontaneous Ca^2+^ oscillations, which arise even in the absence of electrical stimulation. These spontaneous Ca^2+^ spikes are generated by rhythmic Ca^2+^ release from sarcoplasmic reticulum (SR) through RyR2, which in turn evokes a Na^+^/Ca^2+^ exchanger-mediated membrane depolarization and activates VGCCs^25,26^.

**Figure 2:**
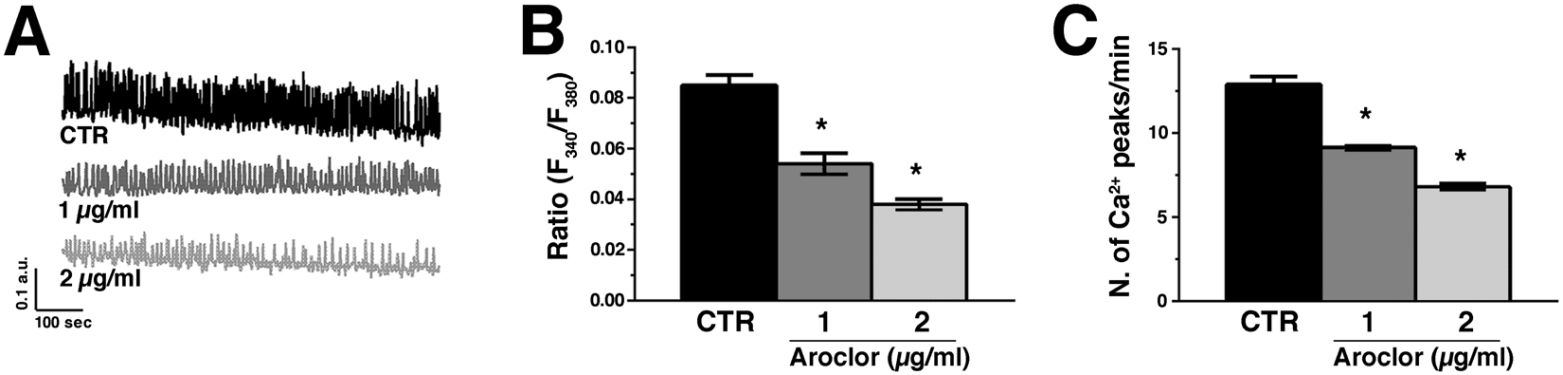
Ca^2+^ oscillations in cardiac beating syncytia. (**A**) Representative tracing of spontaneous Ca^2+^ spikes recorded in CTR or in the presence of either 1 or 2 µg/mL Aroclor. (**B**) Mean ± SE of the amplitude of the Ca^2+^ peaks recorded under the designated treatments. (**C**) Mean ± SE of the frequency of spontaneous Ca^2+^ spikes. *p < 0.05.

When loading beating syncytia with the Ca^2+^-sensitive fluorochrome Fura-2/AM, we observed, compared to CTR unexposed cells, a significant (*p* < 0.05) decrease in both amplitude (Fig. 2A,B) and frequency of spontaneous Ca^2+^ transients (Fig. 2A,C) in cardiomyocytes exposed to 1 or 2 μg/ml Aroclor. These results show that Aroclor may reduce cardiomyocyte contraction properties by interfering with the intracellular Ca^2+^ handling machinery in beating syncytia.

### Aroclor does not alter the molecular machinery underlying spontaneous Ca^2+^ activity

**Figure 3:**
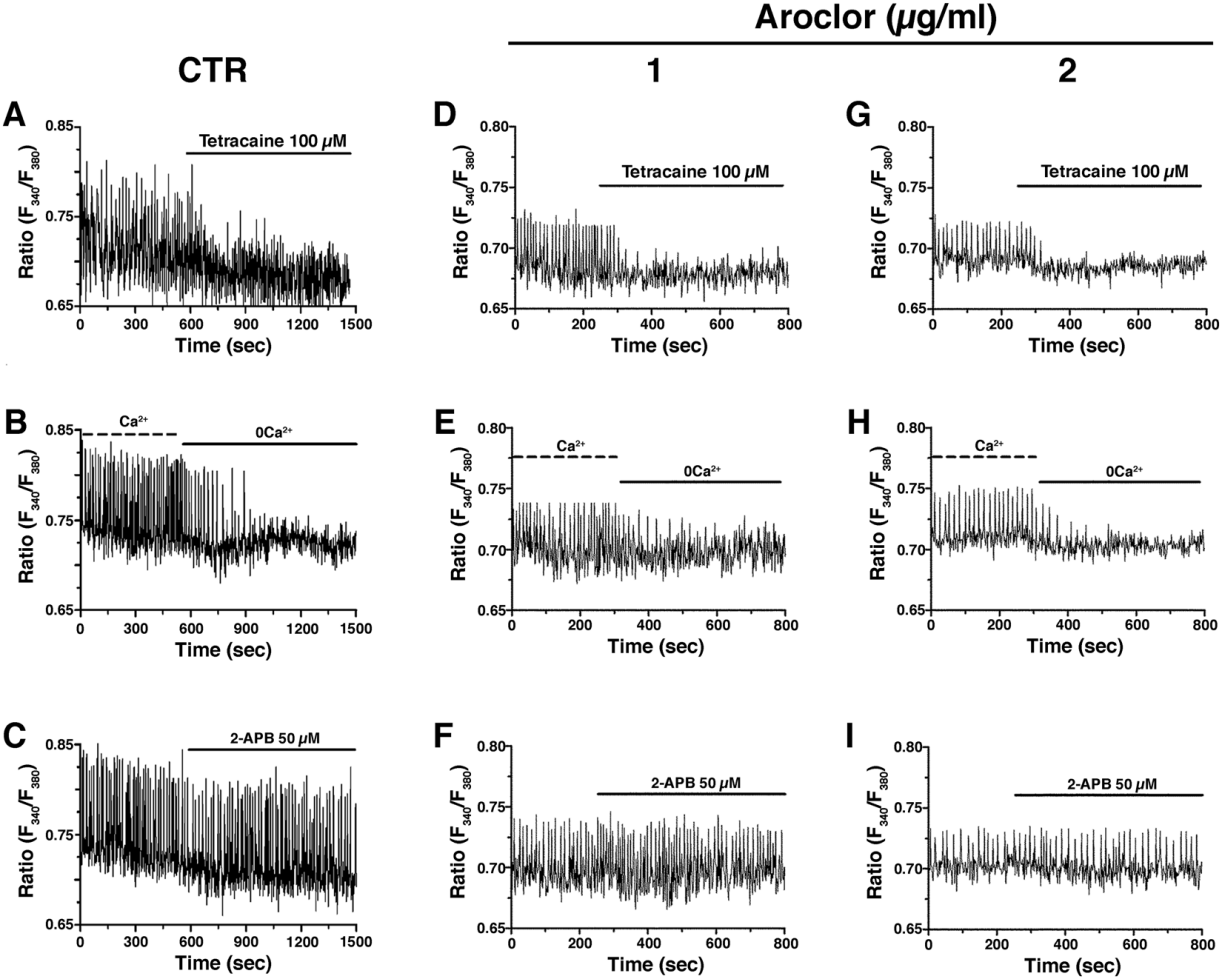
Intracellular Ca^2+^ spikes in CTR and 1 or 2 µg/ml-exposed cardiac beating syncytia. (**A**),(**D**) and (**G**) Tetracaine (100 µM) caused a prompt inhibition of the intracellular Ca^2+^ spikes; (**B**),(**E**) and (**H**) Extracellular Ca^2+^ removal (0Ca^2+^) did not induce the abrupt interruption of the Ca^2+^ transients, but resulted in the progressive decline in the amplitude of the Ca^2+^ transients; (**C**),(**F**) and (**I**) Superfusion of 2-APB (50 µM) did not affect spontaneous Ca^2+^ spikes.

In CTR samples, tetracaine (100 μM), a selective RyR inhibitor^27^, caused a prompt inhibition of the intracellular Ca^2+^ spikes (Fig. 3A), thereby confirming the crucial role played by RyR2 in shaping the Ca^2+^ signal^25,26^. Similar to previous studies^25^, removal of external Ca^2+^ (0Ca^2+^) did not induce the abrupt interruption of the Ca^2+^ transients, but resulted in the progressive decline of their amplitude, determining complete arrest of Ca^2+^ activity after about 5 min (Fig. 3B). This finding is consistent with the notion that Ca^2+^ entry is triggered by spontaneous Ca^2+^ oscillations to refill the SR Ca^2+^ pool^26^. Conversely, the blockade of InsP_3_Rs with 2-APB (50 µM) failed to affect the ongoing Ca^2+^ transients (Fig. 3C). Overall, these findings confirm that RyR2-dependent rhythmical Ca^2+^ release underlies the onset of the spontaneous Ca^2+^ oscillations, whereas voltage-dependent Ca^2+^ entry maintains them over time.

The same results were obtained when the beating syncytia were exposed to 1 (Fig. 3D–F) or 2 μg/ml (Fig. 3G–I) Aroclor. Therefore, RyR2 and VGCCs interact to shape the spontaneous Ca^2+^ oscillations also in Aroclor-exposed syncytia.

Earlier work showed that angiotensin II and endothelin 1 are able to modulate spontaneous Ca^2+^ oscillations in beating syncytia by inducing InsP_3_-induced Ca^2+^ release from the SR^28^. In agreement with these observations, the application of angiotensin II (1 μM) during ongoing oscillations caused a transient enlargement of the first Ca^2+^ spike, followed by a short-lasting reduction in the amplitude of the subsequent Ca^2+^ oscillations (Supplementary Figure 1SA). Conversely, when beating syncytia were exposed to 1 (Supplementary Figure 1SB) or 2 μg/ml (Supplementary Figure 1SC) Aroclor, angiotensin II (1 μM) caused a large Ca^2+^ spike whose decay to the baseline was overlapped by RyR2-dependent spontaneous Ca^2+^ oscillations. Of note, the Ca^2+^ response to angiotensin II was higher in the presence of 2 μM Aroclor. These observations suggest that the InsP_3_-sensitive Ca^2+^ pool is increased in the presence of Aroclor, which further rules out the contribution of InsP_3_Rs to the spontaneous Ca^2+^ oscillations (see Discussion).

Next, we determined how Aroclor interferes with the single components of the Ca^2+^ toolkit involved in the oscillatory signal, reducing spontaneous intracellular Ca^2+^ oscillations.

### Aroclor affects the intracellular Ca^2+^ oscillations reducing the SR Ca^2+^ content

**Figure 4:**
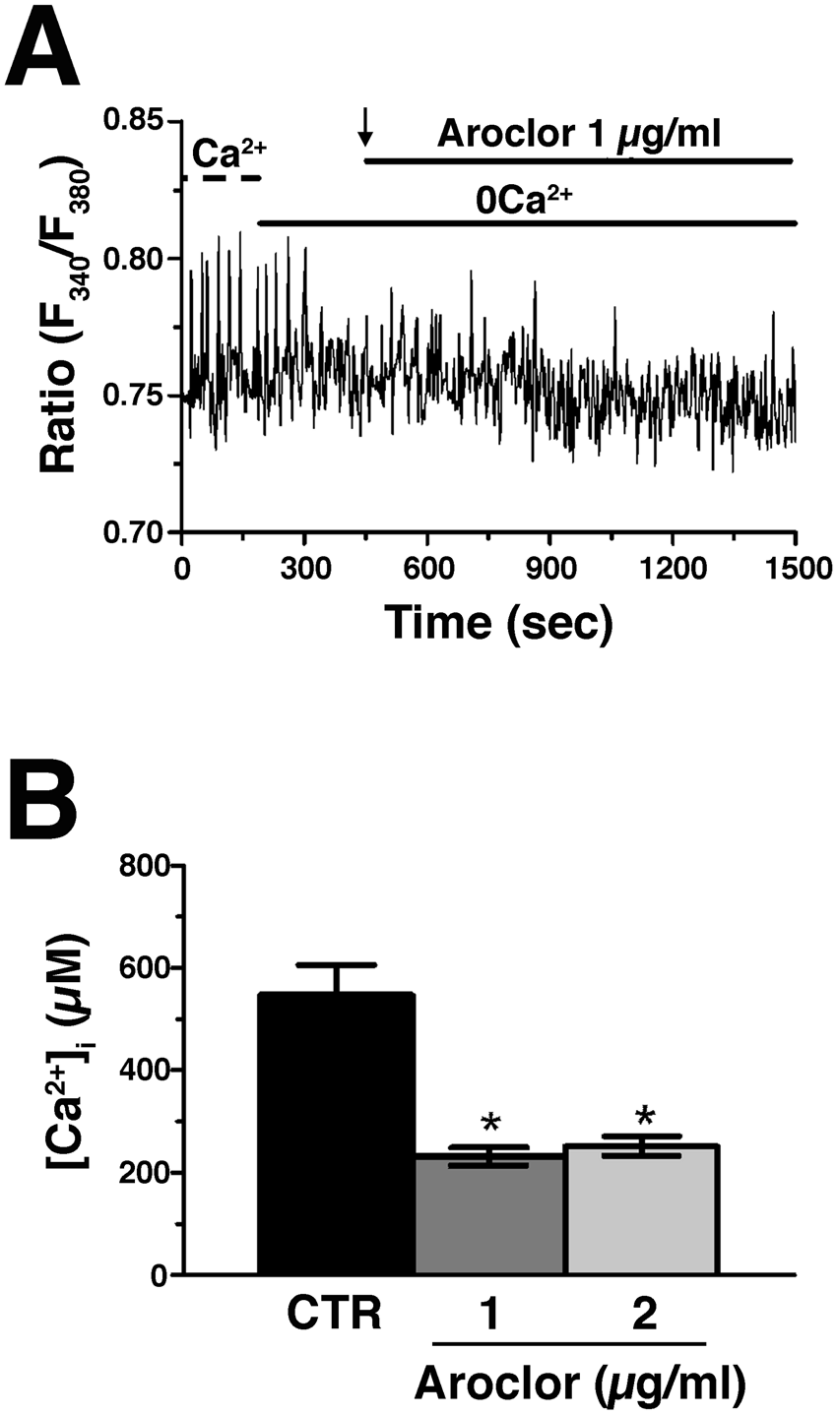
Acute effect of Aroclor on cardiac beating syncytia. (**A**) The acute addition of Aroclor (1 µg/mL, black arrow at 400 s) did not cause intracellular Ca^2+^ mobilization under 0Ca^2+^ conditions. (**B)** Mean ± SE of resting [Ca^2+^]_i_ in cardiac syncytia exposed to either 1 or 2 µg/mL Aroclor for 24 h. *p < 0.05.

**Figure 5:**
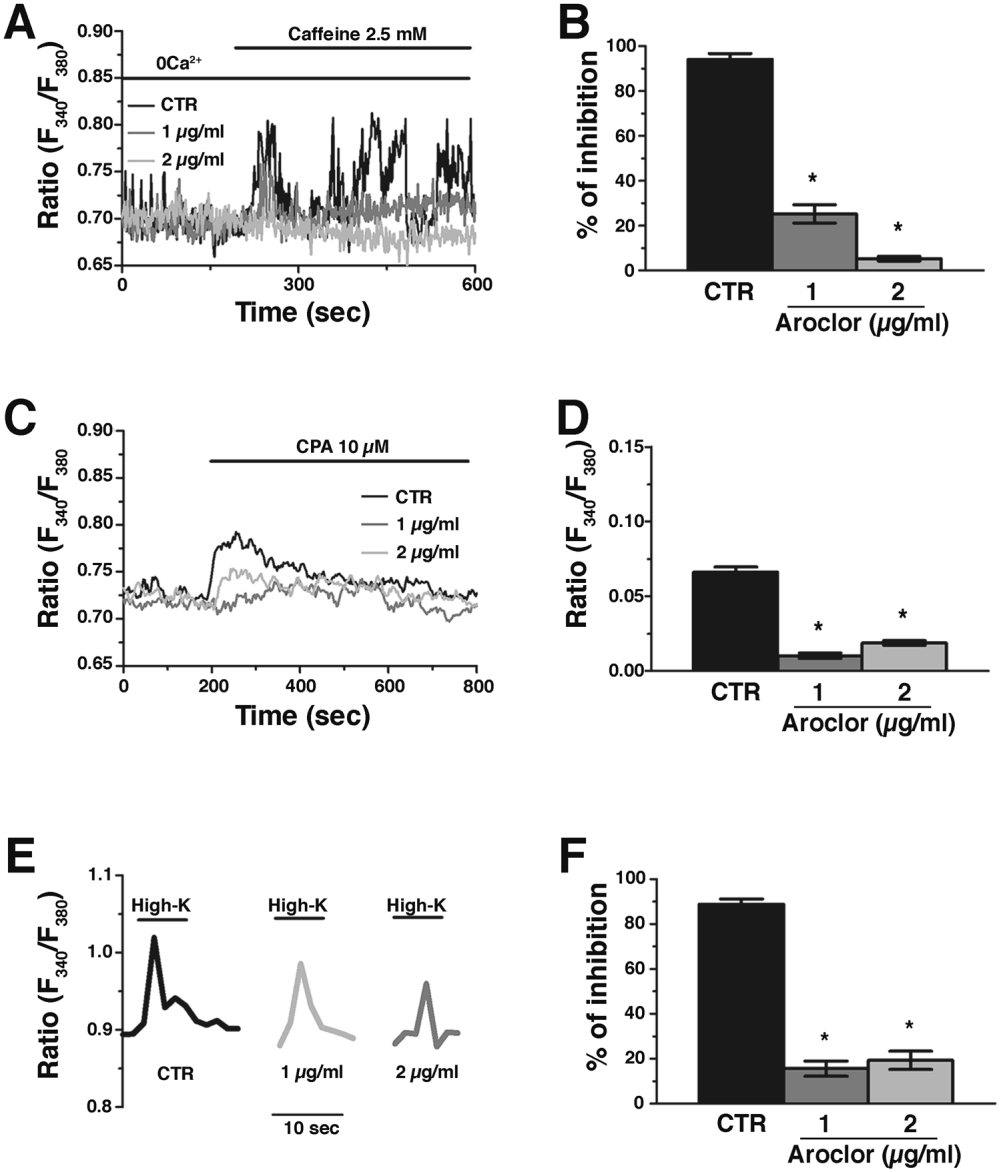
Ca^2+^ entry and RyRs-dependent Ca^2+^ release in 1 or 2 µg/ml-exposed cardiac beating syncytia. (**A**) Representative Ca^2+^ tracings of the Ca^2+^ response to caffeine (2.5 mM) in the absence or in the presence of either 1 or 2 µg/mL Aroclor. Extracellular Ca^2+^ was removed (0Ca^2+^) to prevent any contaminating effect from Ca^2+^ entry. (**B**) Mean ± SE of the percentage of Aroclor-induced inhibition of caffeine-induced Ca^2+^ transients. (**C**) Representative Ca^2+^ tracings of the Ca^2+^ response to cyclopiazonic acid (10 µM; CPA) in the absence or in the presence of either 1 or 2 µg/mL Aroclor. Extracellular Ca^2+^ was removed (0Ca^2+^) to prevent any contaminating effect from Ca^2+^ entry. (**D**) Mean ± SE of the percentage of Aroclor-induced inhibition of CPA-induced Ca^2+^ transients. (**E**) Representative tracings of the Ca^2+^ signals induced by sustained depolarization caused by high KCl (High-K) in the extracellular solution in the absence or in the presence of either 1  or 2 µg/mL Aroclor. (**F**) Mean±SE of the percentage of Aroclor-induced inhibition of High-K-induced Ca^2+^ transients. *p < 0.05.

The acute addition of Aroclor in the 0Ca^2+^ medium did not induce any detectable variation in [Ca^2+^]_i_ at both 1 (Fig. 4A) or 2 μg/ml (not shown). Instead, 24 h exposure to Aroclor caused a significant (*p* < 0.05) reduction in basal [Ca^2+^]_i_ (Fig. 4B). Therefore, we turned our attention to the Ca^2+^-permeable pathways underlying the spontaneous Ca^2+^ oscillations, i.e., RyR2 and VGCCs. Caffeine (20 mM), a selective RyR agonist, triggered a Ca^2+^ response in CTR samples, while it failed in the presence of Aroclor at both concentrations (Fig. 5A,B). To further assess the effect of Aroclor on SR Ca^2+^ content, we used cyclopiazonic acid (CPA), known to inhibit SERCA activity thereby causing the progressive efflux of intraluminal Ca^2+^ through unidentified leakage channels^29,30^. This manoeuvre leads to the rapid depletion of the SR Ca^2+^ pool under 0Ca^2+^ conditions and, therefore, causes a transient increase in [Ca^2+^]_i_. The [Ca^2+^]_i_ indeed returns to the original baseline due to the concerted interaction of the Na^+^/Ca^2+^ exchanger (NCX), plasma-membrane Ca^2+^-ATPase (PMCA) and mitochondria. CPA-induced intracellular Ca^2+^ mobilization is a well-known index of SR Ca^2+^ content and is widely employed to compare SR Ca^2+^ content between control and treated cells^29–31^. In our experiments, exposure to Aroclor caused a significant reduction in CPA-induced Ca^2+^ release under 0Ca^2+^ conditions (Fig. 5C,D), which hints at a remarkable reduction in SR Ca^2+^ levels.

Finally, we monitored voltage-dependent Ca^2+^ entry by depolarizing cardiomyocytes with high-KCl solution. We found that high-KCl-induced Ca^2+^ entry was significantly (*p* < 0.05) reduced in the presence of each dose of Aroclor (Fig. 5E,F).

Overall, these data strongly suggest that Aroclor reduces the SR Ca^2+^ content, thereby impairing RyR2-dependent Ca^2+^ release, decreases the resting [Ca^2+^]_i._ and inhibits voltage-dependent Ca^2+^ inflow in cardiomyocytes. As a consequence, the spontaneous Ca^2+^ oscillations that drive cardiac contraction are inhibited, which contributes to explain Aroclor-dependent cardiac functional alterations.

### Aroclor modifies the expression of genes involved in Ca^2+^ toolkit

**Figure 6:**
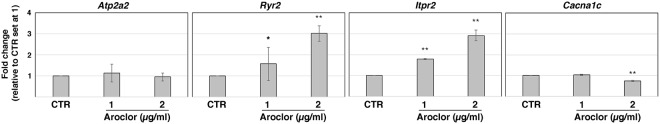
Gene expression analysis of cardiac beating syncytia. Expression profiles of *Atp2a2*, *Ryr2*, *Itpr2* and *Cacna1c*. The expression values of CTR samples were set at 1 for the calculation of the *n*-fold change. Values are expressed as mean ± SD. *p < 0.05; **p < 0.001.

To assess whether Aroclor-induced inhibition of spontaneous Ca^2+^ oscillations is associated with changes in the expression profile of Ca^2+^ toolkit genes, we analyzed *Atp2a2* (coding for SERCA2A, cardiac isoform of the Ca^2+^-ATPase that sequesters Ca^2+^ back into the SR), *Ryr2* (cardiac muscle SR RyR isoform), *Cacna1c* (α subunit of L-type VGCCs) and *Itpr2* (cardiac muscle SR InsP_3_R isoform). The exposure to both Aroclor concentrations did not significantly alter the quantitative expression of *Atp2a2* gene (*p* > 0.05). Conversely, the expression of *Ryr2* and *Itpr2* significantly increased (1.7- and 1.9-fold, respectively), whereas the expression of *Cacna1c s*ignificantly decreased (0.7-fold) only in syncytia exposed to 2 µg/ml (Fig. 6).

## Discussion

Our study demonstrates that Aroclor exerts its detrimental effects on the beating properties of perinatal-like cardiomyocytes, acting on the intracellular Ca^2+^ machinery. Rhythmic beating in foetal and neonatal cardiomyocytes depends on spontaneous events of RyRs-mediated Ca^2+^ release from the SR, which may in turn evoke small depolarizations that trigger L-type VGCCs-driven action potentials^25,26^. The spontaneous intracellular Ca^2+^ oscillations represent the main source of Ca^2+^ for the contractile machinery during cardiac development^26,32^. It has long been known that PCB toxicity is consequent to the alteration of the intracellular Ca^2+^ machinery^10,33^.

Consistent with these earlier observations, Aroclor heavily impairs spontaneous intracellular Ca^2+^ oscillations of cardiomyocytes, affecting both their frequency and amplitude. The signalling machinery underlying the rhythmic Ca^2+^ signal was not affected by the treatment, as, both in the absence and in the presence of Aroclor 1254, the intracellular Ca^2+^ oscillations were mainly driven by RyRs and supported by extracellular Ca^2+^ entry. We observed that, both in the absence or in the presence of Aroclor, the repetitive Ca^2+^ spikes were abruptly inhibited by tetracaine, thereby confirming that RyRs drive the spontaneous Ca^2+^ oscillations, and the removal of extracellular Ca^2+^ did not cause a prompt interruption of the Ca^2+^ spikes, as expected if they were mainly sustained by intracellular Ca^2+^ mobilization. Also, the pharmacological blockade of InsP_3_Rs with 2-APB, a powerful and reliable blocker of such receptors^34,35^, never affected the spontaneous Ca^2+^ oscillations, thereby ruling out InsP_3_Rs from the mechanisms that drive the beating activity under our conditions. Of note, the stimulation of InsP_3_Rs with angiotensin II caused a larger increase in [Ca^2+^]_i_ in beating syncytia exposed to Aroclor, while CTR cardiomyocytes only showed a modest enlargement of the first Ca^2+^ spike arising after agonist addition followed by a transient reduction in the amplitude of the following Ca^2+^ oscillations. These data strongly suggest that: 1) InsP_3_Rs do not play a prominent role in inducing the spontaneous Ca^2+^ spikes, as the InsP_3_-sensitive SR Ca^2+^ pool is up-regulated by Aroclor exposure, while the amplitude and frequency of Ca^2+^ oscillations are reduced; and 2) InsP_3_Rs are, however, able to modulate RyRs-dependent Ca^2+^ release, as the transient depletion of the InsP_3_-sensitive SR Ca^2+^ pool leads to a short-lasting decrease in the amplitude of Ca^2+^ spikes in CTR cardiomyocytes. The increase in *Itpr2* expression could explain the largest Ca^2+^ signal induced by angiotensin II in the presence of Aroclor. Consistently, 2 μg/ml Aroclor induces a dose-dependent elevation in both *Itpr2* transcripts and in the peak Ca^2+^ response to angiotensin II.

These data indicate that RyRs and VGCCs work to shape the spontaneous Ca^2+^ oscillations also in Aroclor-exposed syncytia and suggest that the signaling machinery underlying the spontaneous Ca^2+^ signal was not affected by the treatment. Thus, we moved our attention to single components of the Ca^2+^ toolkit involved in the oscillatory signal to evaluate whether and how Aroclor interferes with them. We determined that Aroclor exposure induced a significant reduction in basal [Ca^2+^]_i_ and in SR Ca^2+^ levels in cardiomyocytes, which was reflected in a dramatic decrease in the caffeine-releasable Ca^2+^ pool. Additionally, Aroclor heavily inhibited voltage-dependent Ca^2+^ entry, as measured in response to high-KCl extracellular solution. Therefore, Aroclor affects both the components of the Ca^2+^ toolkit which deliver Ca^2+^ to the cytosol during the spiking activity. Similar results were obtained in neuronal cells, in which it was demonstrated that Aroclor 1254 induced a depletion of SR Ca^2+^ store through the activation of InsP_3_Rs^36,37^.

The acute addition of Aroclor did not trigger any detectable increase in [Ca^2+^]_i_, suggesting that Aroclor is not able to cause a massive and rapid release of intraluminally stored Ca^2+^ which is the most intuitive mechanism to explain the depletion of the SR Ca^2+^ pool underpinning the rhythmic Ca^2+^ release (as discussed in more detail below). This result differs compared to that observed in rodent neurons^38–40^ and PC12 cells^41^, in which PCB-95 and Aroclor 1254 stimulate RyRs-dependent intracellular Ca^2+^ release. It has been demonstrated that PCB congeners sensitize RyRs by binding to one or more components of the RyR macromolecular complex, which could therefore act as receptors^10,42,43^. We speculate that in perinatal cardiomyocytes, characterized by an immature SR^44,45^, the lack of expression of components of the adult RyR macromolecular complex may explain why Aroclor fails to increase [Ca^2+^]_i_. As Aroclor did not evoke an acute Ca^2+^ response, we reasoned that effects observed after 24h-exposure could be explained by an interference with the Ca^2+^ handling machinery.

Real Time PCR analysis demonstrated that both 1 or 2 μg/ml Aroclor induced a significant increase of the expression of *Ryr2*, as reported also in previous studies on rat cerebellum cells^46^. The upregulation of *Ryr2* transcript expression is predicted to increase, rather than decreasing, the release of Ca^2+^ from SR to the cytoplasm and thus increases the spiking activity of beating syncytia. However, it has been reported that Aroclor 1254 at 6–9 μM inhibits SR Ca^2+^ uptake, interfering with SERCA activity^47,48^, and at 10–13 μM severely compromises the SERCA-mediated uptake of Ca^2+^ by SR microsomes^48^. The Aroclor concentrations that we used, i.e. 1 and 2 μg/ml, fall within this range and, therefore, might be able to interfere with SERCA-mediated Ca^2+^ sequestration. This mechanism could explain the fall in SR Ca^2+^ levels and, consequently, in the caffeine-releasable Ca^2+^ pool that we observed in the presence of both 1 and 2 μg/ml Aroclor. Additionally, as SR Ca^2+^ continuously leaks out in the cytosol to maintain the [Ca^2+^]_i_^49^, this model would also explain the drop in resting Ca^2+^ levels caused by Aroclor-treated in the beating syncytia. Future investigations are required to confirm this hypothesis and to understand how Aroclor 1254 interacts with SERCA2A. Our data, however, rule out the down-regulation of *Atp2a2* expression, that codes for SERCA2A, as the transcript levels of this gene are not altered after 24 h Aroclor exposure. The mechanism whereby Aroclor inhibits SERCA activity is yet to be elucidated^47,48^. However, in cardiomyocytes, SERCA activity is severely reduced by cytosolic and mitochondria-derived reactive oxygen species (ROS), which therefore lead to a dramatic reduction in SR Ca^2+^ concentration^50,51^. Interestingly, a recent work showed that ROS production through the mitochondrial pathway started to increase after 1 h of Aroclor exposure in the human lung cancer cell line A549^52^. This mechanism could explain why the inhibitory effect of Aroclor is not acute and requires a prolonged incubation also in the beating syncytia.

In addition to reducing SR Ca^2+^ levels, we found that Aroclor attenuated voltage-dependent Ca^2+^ entry, which is necessary to maintain the spontaneous Ca^2+^ oscillations over time, at both 1 and 2 μg/ml. Real Time PCR disclosed that only 2 μg/ml Aroclor 1254 decreased the expression of *Cacna1c*, which encodes for the α subunit of L-type VGCCs. While this mechanism is likely to play a major role in reducing voltage-dependent Ca^2+^ entry at the higher dose, it does not explain the inhibitory effect observed at 1 μg/ml. However, earlier reports revealed that PCBs are able to increase Ca^2+^ permeability across the plasma membrane in several cell types, causing a long-lasting influx of Ca^2+^ through unidentified Ca^2+^-permeable channels^53^ or through store-operated Ca^2+^ channels^54^. Also, Aroclor 1254 activates the Ca^2+^-permeable *N*-methyl-D-aspartate receptors^55^, inhibits the non-selective cation channel TRP Vanilloid 6 (TRPV6)^56^ and stimulates Cl^–^permeable γ-aminobutyric acid (GABA)_A_ receptors^55,57^ in newborn rat neocortical neurons. All together, these findings clearly demonstrate that PCB congeners, including Aroclor 1254, have the potential to modulate plasma membrane channels, although it remains to be elucidated whether this interaction is direct or mediated by auxiliary partners. We hypothesize that, besides reducing *Cacna1c* expression, Aroclor 1254 inhibits L-type VGCCs through a non-yet identified mechanism in cardiomyocytes.

In conclusion, for the first time, we demonstrate that perinatal cardiac syncytia exposed to Aroclor 1254 undergo a dramatic functional alteration of the kinematics contractile properties, due to the disruption of the intracellular Ca^2+^ machinery. These findings contribute to the understanding of the molecular underpinnings of PCBs-induced cardiovascular alterations, which are emerging as an additional life-threatening hurdle associated to PCB pollution. Therefore, PCBs-dependent alteration of intracellular Ca^2+^ machinery is the most likely trigger of developmental cardiac functional alterations.

## Methods

### Cell lines

R1 mESC line (kindly provided by Dr. Nagy from Samuel Lunenfeld Research Institute, Mount Sinai Hospital, Toronto, Ontario, Canada) and STO cell line (ATCC, CRL-2225) were cultivated as previously described^58^. Briefly, R1 was cultivated in Knockout DMEM added with 15% ESC Qualified FBS, 2 mM L-glutamine, 1X non-essential amino acids, 0.5% penicillin/streptomycin (all from Thermo Fisher Scientific), 0.1 mM β-mercaptoethanol (Sigma) and 500 U/ml ESGRO-LIF (Merck Millipore, Italy). The STO cell line was maintained in DMEM (Sigma) supplemented with 10% foetal bovine serum, 4 mM L-glutamine, 1X non-essential amino acids, 0.5% penicillin–streptomycin solution (all from Thermo Fisher Scientific), 0.1 mM β-mercaptoethanol (Sigma) and 0.2 mg/mL geneticin (Sigma). ESCs were routinely passaged enzymatically every 2/3 days with trypsin/EDTA 0.05%, alternating a passage on STO feeder cells with two passages on gelatin-coated p55 dish, and maintained in an incubator at 37 °C with 5% CO_2_ in air.

### Differentiation of mESCs into perinatal-like cardiomyocytes and Aroclor treatment

mESCs were induced to differentiate through the formation of embryoid bodies (EBs) *in vitro* by removing the Leukemia Inhibitory Factor (LIF) from culture medium (differentiation medium), using the hanging drop method^59,60^. For EBs formation, about seventy 20 µl droplets of differentiation medium containing 10^3^ mESCs were plated on the lid of p55 Petri dishes. On day 3 of culture, the developing EBs were transferred on 0.1% agarose-coated tissue dishes (Corning) and from day 5, about 5–8 EBs were plated in single 1.9 cm^2^ well and cultivated up to 15 days.

Aroclor 1254 (Pancreac Nova Chimica) was dissolved in 100% dimethyl sulfoxide (DMSO; Sigma) to a concentration of 20 mg/ml. This solution was added to the differentiation medium on day 15 to a final concentration of 1 or 2 µg/ml. As control (CTR) samples, cells were exposed to 0.01% DMSO. Cells were harvested 24 h after either Aroclor or DMSO exposure. This procedure was repeated for three independent experiments.

### Contraction assay

On day 5 of differentiation, 25 EBs were plated onto 22 mm gelatin-coated Glass Bottom Dish (WillCo Wells) and cultured up to day 15. At day 15, cells were exposed to Aroclor and then transferred into the culture chamber of a Nikon BioStation IM at 37 °C and 5% CO_2_ for video recording. For each of the three independent experiments, AVI videos of the beating syncytia were recorded from 10 randomly chosen CTR or Aroclor-exposed samples, using the Snagit software and further analyzed with the Video Spot Tracker (VST) program (http://cismm.cs.unc.edu/downloads). Then, videos were processed according to the image processing algorithm based on the Matlab programming language (The MathWorks, Inc., Natick, MA)^22,61^.

### Measurement of Ca^2+^ transients in mESCs-derived cardiomyocytes

Ca^2+^ dynamics in mESCs-derived cardiomyocytes were measured by using a conventional epifluorescence Ca^2+^ imaging system, as shown elsewhere^25,26^. At day 5 of differentiation, EBs were plated on 12 mm coverslips in 24-multiwell plates. At day 15 beating cardiac syncytia, exposed to DMSO, 1 or 2 µg/ml Aroclor for 24 h, were analyzed for Ca^2+^ transients. Cells were loaded with 0.5 μM Fura-2/AM (1 mM stock in DMSO; Thermo Fisher Scientific) for 20 min at 37 °C. Then, after washing with pre-warmed Ca^2+^-containing Tyrode’s solution (NaCl 154 mM, KCl 4 mM, MgCl_2_ 1 mM, CaCl_2_ 2 mM, HEPES 5 mM and Glucose 5.5 mM), the coverslips were fixed to the bottom of a Petri dish and the cells observed by an upright epifluorescence Axiolab microscope (Carl Zeiss, Oberkochen, Germany), equipped with a Zeiss 40× Achroplan objective (water-immersion, 2.0 mm working distance, 0.9 numerical aperture). Voltage-dependent Ca^2+^ entry was stimulated by replacing 40 mM NaCl with an equimolar amount of KCl (high-KCl solution). Intracellular Ca^2+^ release was monitored in the medium without Ca^2+^ (0 Ca^2+^) and supplemented with 10 mM EGTA. Cells were excited alternately at 340 and 380 nm wavelengths and the emitted light was detected at 510 nm. A first neutral density filter (1 or 0.3 optical density) reduced the overall intensity of the excitation light and a second neutral density filter (optical density = 0.3) was coupled to the 380 nm filter to reach the intensity of the 340 nm wavelength. A round diaphragm was used to increase the contrast. Excitation filters were mounted on a filter wheel (Lambda 10, Sutter Instrument, Novato, CA, USA). Custom software, working in LINUX environment, was used to drive the camera (Extended-ISIS Camera, Photonic Science, Millham, UK), the filter wheel and to measure and plot on-line the fluorescence from 30–45 rectangular “regions of interest” (ROI) enclosing 30–60 areas within each of the beating syncytia. Each ROI was identified by a number. The ratio of fluorescence emitted at 340 and 380 nm was recorded as an indicator of the changes in intracellular Ca^2+^ concentration ([Ca^2+^]_i_). The experiments were performed at room temperature (22 °C). Ratio measurements were performed and plotted every 1.5 s for 900 s. The resting [Ca^2+^]_i_ was measured by exploiting the Grynkiewicz equation, as shown in Zuccolo *et al*.^30^.

### RNA extraction, reverse transcription and quantitative Real-Time PCR

On day 16, following 24 h Aroclor exposure, RNA was extracted using the GenElute Mammalian Total RNA Kit according to the manufacturer’s instruction (Sigma) from about 200 EBs from each CTR and 1 or 2 µg/ml Aroclor-exposed samples. Three independent experiments were performed on a total of about 1800 EBs.

Reverse transcription was performed in a final volume of 20 µl reaction mixture with 1 µg of RNA, 1x PCR buffer, 5 mM MgCl_2_, 4 mM of each dNTP, 0.625 M oligo d(T)_16_, 1.875 M Random Hexamers, 20 U RNase Inhibitor, 50 U MuLV reverse transcriptase (all from Thermo Fisher Scientific). The conditions for the reverse transcription were as follows: 25 °C for 10 min, 42 °C for 15 min, 99 °C for 5 min. One twentieth of the resulting cDNA was amplified in duplicate by Real-Time PCR in 20 µl reaction mixture with 200 nM of each specific primer (designed using Primer 3 software; see Table 1S) and the MESA GREEN qPCR MasterMix Plus for SYBR assay no ROX sample (Eurogentec) at 1X as final concentration. The amplification reaction, performed in a Rotorgene 6000 (Corbett Life Science), was with the following program: 95 °C for 5 min, followed by 40 cycles at 95 °C for 10 s, 60 °C for 15 s, 72 °C for 20 s. β-2-microglobulin gene expression was used for sample normalization^60^. The Rotorgene 6000 Series Software 1.7 was used for the comparative concentration analysis.

### Statistics

PCR expression data are presented as means ± standard deviation (SD), while the syncytium contractile parameters are expressed as means ± 95% confidence interval for the differences between means. Data were analyzed by the one-way ANOVA and by the *post hoc* LSD test. As to Ca^2+^ imaging data, the frequency of spontaneous Ca^2+^ transients was evaluated by dividing the number of Ca^2+^ transients arising over the stabilization of intracellular Ca^2+^ dynamics (i.e. after 1–3 min from the beginning of the recording) by 60 s. The amplitude of intracellular Ca^2+^ release in response to CPA, which is commonly used to estimate SR Ca^2+^ content, or caffeine and the amplitude of high-KCl-induced Ca^2+^ entry was measured as the difference between the ratio at the peak of intracellular Ca^2+^ mobilization and the mean ratio of 1 min baseline before the peak. Pooled data are given as means ± standard error (SE) and statistical significance (*p* < 0.05) was evaluated by the Student’s *t*-test for unpaired observations. Each Ca^2+^ trace is representative of 80–200 ROIs recorded from at least three embryoid bodies.

## Electronic supplementary material

Supplementary Information

Supplementary Video 1

Supplementary Video 2

Supplementary Video 3
