# Supplementary material for: Polychlorinated biphenyls reduce the kinematics contractile properties of embryonic stem cells-derived cardiomyocytes by disrupting their intracellular Ca2+ dynamics
Source: Sci Rep. 2018 Dec 17;8:17909. doi: 10.1038/s41598-018-36333-z (PMC6297156; doi:10.1038/s41598-018-36333-z)
Supplement: Supplementary file 1 — Supplementary Information [file 41598_2018_36333_MOESM1_ESM.docx]

**Polychlorinated biphenyls reduce the kinematics contractile properties of embryonic stem cells-derived cardiomyocytes by disrupting their intracellular Ca^2+^ dynamics**

Paola Rebuzzini^1,2*^, Estella Zuccolo^3^, Cinzia Civello^1^, Lorenzo Fassina^2,4^, Juan Arechaga^5^, Amaia Izquierdo^5^, Pawan Faris^3,6^, Maurizio Zuccotti^1,2*^, Francesco Moccia^3^ and Silvia Garagna^1,2*^

^1^Laboratorio di Biologia dello Sviluppo, Dipartimento di Biologia e Biotecnologie, Università degli Studi di Pavia, Italy;

^2^Centre for Health Technologies (C.H.T.), Università degli Studi di Pavia, Italy;

^3^Laboratorio di Fisiologia Generale, Dipartimento di Biologia e Biotecnologie, Università degli Studi di Pavia, Italy;

^4^Dipartimento di Ingegneria Industriale e dell’Informazione, Università degli Studi di Pavia, Italy;

^5^Laboratory of Stem Cells, Development and Cancer, Department of Cell Biology and Histology, Faculty of Medicine and Nursing, Universidad del País Vasco, Spain;

^6^Department of Biology, College of Science, Salahaddin University, Erbil, Kurdistan-Region of Iraq, Iraq.

*Corresponding authors:

Paola Rebuzzini

Laboratorio di Biologia dello Sviluppo

Dipartimento di Biologia e Biotecnologie ‘Lazzaro Spallanzani’

Università degli Studi di Pavia

Via Ferrata 9, 27100 Pavia, Italy

Tel +39 0382 986323

Fax +39 0382 986270

e-mail: [paola.rebuzzini@unipv.it](mailto:paola.rebuzzini@unipv.it)

Silvia Garagna

Laboratorio di Biologia dello Sviluppo

Dipartimento di Biologia e Biotecnologie ‘Lazzaro Spallanzani’ and “Centre for Health Technologies”

Università degli Studi di Pavia

Via Ferrata 9, 27100 Pavia, Italy

Tel +39 0382 986323

Fax +39 0382 986270

e-mail: silvia.garagna@unipv.it

Maurizio Zuccotti

Laboratorio di Biologia dello Sviluppo

Dipartimento di Biologia e Biotecnologie ‘Lazzaro Spallanzani’

Università degli Studi di Pavia

Via Ferrata 9, 27100 Pavia, Italy

Tel +39 0382 986323

Fax +39 0382 986270

e-mail: maurizio.zuccotti@unipv.it

**SUPPLEMENTARY**

**Table 1S.** Oligonucleotides used for Real Time PCR amplification.

| **Gene** | **Primer forward** | **Primer Reverse** | **Amplicon length (bp)** |
| --- | --- | --- | --- |
| *Atp2a2* | 5’ CAAGCATACTGACCCTGTCCC 3’ | 5’ CACCACCACTCCCATAGCTTT 3’ | 109 |
| *Itpr2* | 5’ TTCCTGCAGACGATCGTGAAA 3’ | 5’ ATATCAACACATCCTCGCCCC 3’ | 100 |
| *Ryr2* | 5’ TATGCCTTCTACCCACTCCTGA 3’ | 5’ TCAGCCACCATTCGAAATAGCT 3’ | 104 |
| *Cacna1c* | 5’ GAAGAAGCCGAGTACTGCAGT 3’ | 5’ TCTCCCTCTTGTCCTCCTCTG 3’ | 109 |
| *β2m* | 5’ GAATTCACCCCCACTGAGACT 3’ | 5’ TGCTTGATCACATGTCTCGAT 3’ | 103 |

**Legends to Videos**

**Video 1S.** Video of beating syncytia, recorded from a CTR sample.

**Video 2S.** Video of beating syncytia, recorded from a sample after 24h exposure to 1 µg/ml Aroclor.

**Video 3S.** Video of beating syncytia, recorded from a sample after 24h exposure to 2 µg/ml Aroclor.





**Figure 1S. Angiotensin-II effect on cardiac beating syncytia.** **A.** Representative tracing of the effect of angiotensin-II (1 μM) on ongoing Ca^2+^ oscillations in control cardiomyocytes. **B.** Representative tracing of the effect of angiotensin-II (1 μM) on ongoing Ca^2+^ oscillations in cardiomyocytes exposed to 1 μg/ml Aroclor 1254. **C.** Representative tracing of the effect of angiotensin-II (1 μM) on ongoing Ca^2+^ oscillations in cardiomyocyte exposed to 2 μg/ml Aroclor 1254. Please, note that angiotensin-II enlarged the duration of the first Ca^2+^ transient recorded under control conditions (**A**), while it caused 1 or 2 Ca^2+^ spikes in the presence of 1 (**B**) and 2 μg/ml (**C**) Aroclor 1254. The largest response to angiotensin II in exposed beating syncytia is likely to be due to the up-regulation of *Itpr2* induced by Aroclor.
